# BCL-w: apoptotic and non-apoptotic role in health and disease

**DOI:** 10.1038/s41419-020-2417-0

**Published:** 2020-04-21

**Authors:** Mariusz L. Hartman, Malgorzata Czyz

**Affiliations:** Department of Molecular Biology of Cancer, Medical University of Lodz, 6/8 Mazowiecka Street, 92-215 Lodz, Poland

**Keywords:** Oncogenes, Apoptosis, Cell invasion, Senescence, Molecular neuroscience

## Abstract

The BCL-2 family of proteins integrates signals that trigger either cell survival or apoptosis. The balance between pro-survival and pro-apoptotic proteins is important for tissue development and homeostasis, while impaired apoptosis contributes to several pathologies and can be a barrier against effective treatment. BCL-w is an anti-apoptotic protein that shares a sequence similarity with BCL-X_L_, and exhibits a high conformational flexibility. BCL-w level is controlled by a number of signaling pathways, and the repertoire of transcriptional regulators largely depends on the cellular and developmental context. As only a few disease-relevant genetic alterations of *BCL2L2* have been identified, increased levels of BCL-w might be a consequence of abnormal activation of signaling cascades involved in the regulation of BCL-w expression. In addition, BCL-w transcript is a target of a plethora of miRNAs. Besides its originally recognized pro-survival function during spermatogenesis, BCL-w has been envisaged in different types of normal and diseased cells as an anti-apoptotic protein. BCL-w contributes to survival of senescent and drug-resistant cells. Its non-apoptotic role in the promotion of cell migration and invasion has also been elucidated. Growing evidence indicates that a high BCL-w level can be therapeutically relevant in neurodegenerative disorders, neuron dysfunctions and after small intestinal resection, whereas BCL-w inhibition can be beneficial for cancer patients. Although several drugs and natural compounds can bi-directionally affect BCL-w level, agents that selectively target BCL-w are not yet available. This review discusses current knowledge on the role of BCL-w in health, non-cancerous diseases and cancer.

## Facts

- In addition to its pro-survival function, BCL-w plays a non-apoptotic role in regulation of cell motility and senescence.
- The role of BCL-w has been demonstrated in many types of normal cells and diseases, including disorders of nervous system and cancer.
- A plethora of regulators involved in the control of *BCL2L2* expression determine cellular and developmental contexts of BCL-w level and activity.

## Open questions

- How unique is the apoptotic and non-apoptotic role of BCL-w compared with other members of the BCL-2 family of proteins?
- Can BCL-w level be a prognostic factor in cancer and non-cancerous diseases?
- Can BCL-w be selectively targeted by natural and/or synthetic drugs?

## Introduction

**Fig. 1:**
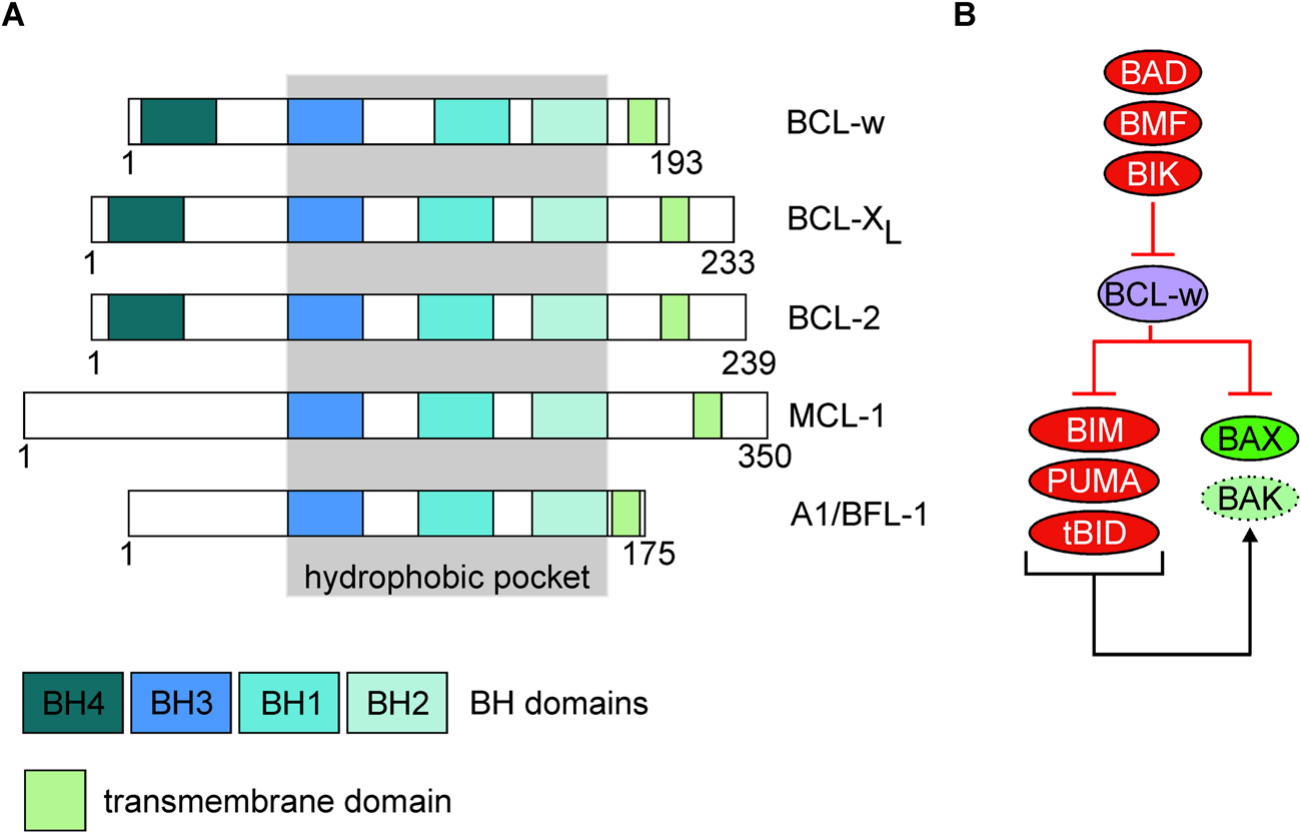
BCL-w is an anti-apoptotic member of the BCL-2 family of proteins. **a** Schematic domain structure of BCL-w and other pro-survival members of the BCL-2 family. Number of amino acid residues in particular human BCL-2-like protein is shown below. BH3 homology (BH) and transmembrane domains are shown in different colors, and BH domains involved in the formation of a hydrophobic pocket are marked in gray background. **b** The functional relationship between BCL-w and pro-apoptotic members of BCL-2 family.

The balance between pro-survival and pro-apoptotic proteins is important for tissue development and homeostasis, while impaired apoptosis contributes to several pathologies and can be a barrier against effective treatment^1,2^. Proteins from the B-cell lymphoma-2 (BCL-2) family are essential integrators of signals that trigger cell survival or apoptosis, while cell fate depends on the abundance, localization, and interactions between particular BCL-2-like proteins^3^. The BCL-2 family members are classified based on the structure and structure-related function. Anti-apoptotic members of this family, BCL-2 itself, B-cell lymphoma-extra-large (BCL-X_L_), B-cell lymphoma-w (BCL-w), BCL-2-related protein A1/BCL-2-related isolated from fetal liver-11 (A1/BFL-1) and myeloid cell leukemia-1 (MCL-1)^4,5^ share four BCL-2-homology (BH) domains (BH1-BH4), but A1/BFL-1 and certain isoforms of MCL-1 lack the BH4 domain (Fig. 1a)^6^. The pro-apoptotic proteins such as BCL-2-associated X protein (BAX) and BCL-2 antagonist/killer (BAK) possess BH1-BH3 motifs^4,5,7^. In addition to their non-canonical roles^8,9^, BAX and BAK directly execute mitochondrial outer membrane permeabilization (MOMP), which is usually considered a point of no return in an apoptotic cascade^10^. The proteins of the third subclass (BH3-only proteins) share exclusively the BH3 domain. BCL-2-interacting mediator of cell death (BIM), p53-upregulated modulator of apoptosis (PUMA) and truncated form of BH3-interacting domain death agonist (tBID) are called ‘activators’ as they can bind to and provoke a conformational change of BAX and/or BAK to induce MOMP. In turn, BH3-only proteins that do not associate with BAX and BAK are named ‘sensitizers’^5,11,12^. Regulation of apoptosis by the proteins of BCL-2 family relies on the balance between the activity of anti-apoptotic proteins that leash the ‘activators’ and MOMP-initiating molecules, and the ‘sensitizers’ that antagonize the pro-survival members by liberating BAX/BAK and the BAX/BAK-activating BH3-only proteins (Fig. 1b)^4,5^. This succinct review will address the current understanding of the structure and function of BCL-w and its apoptotic and non-apoptotic role in health and disease.

## BCL-w as a pro-survival member of the BCL-2 family of proteins

BCL-w is an anti-apoptotic protein that shares the highest sequence similarity (51%) with BCL-X_L_ in comparison to other pro-survival molecules^13^. BCL-w interacts with BAX and BAK, and several BH3-only proteins such as BAD, tBID, BIM, PUMA, BMF, and BIK as shown by co-immunoprecipitation^14–16^. Results of the isothermal titration calorimetry indicate a preferential binding of BCL-w to BAX in comparison to its binding to BAK, with *K*_D_ = 22.9 nM and *K*_D_ = 114 nM, respectively^17^. The precisely regulated interactions between pro- and anti-apoptotic proteins are possible due to the spatial architecture of the BH1-3 domains. They form the hydrophobic groove responsible for the sequestration capability of pro-survival molecules (Fig. 1a), and structure of the binding site dictates the repertoire of interacting proteins^4,5^. For example, it has been demonstrated that a Gly94 residue within the BH1 domain of BCL-w is critical for BAX inhibition^18^, and a G94E substitution in BCL-w abolishes its cytoprotective function in response to interleukin-3 (IL-3) deprivation^14^. In addition, FXXRXR and R/KXV/IXF motifs in BCL-w enables interaction with protein phosphatase 1α (PP1α)^19^. Consequently, BCL-w forms a complex with PP1α and BAD, which leads to dephosphorylation of BAD upon interleukin-4 (IL-4) deprivation^19^. The interactions between BCL-w and poorly characterized members of the BCL-2 family, BFK^20^ and BOP^21^ have also been reported. Moreover, α1/2 and α5/6 loops of BCL-w can associate with p53 through p53 DNA-binding domain, which contributes to transcription-independent regulation of cell death^22^. BCL-w also interacts with BH3-like domain of Beclin-1, an autophagy-related protein^23^. Interactions between anti- and pro-apoptotic proteins can be precisely quantified as recently demonstrated by using a fluorescence resonance energy transfer (FRET) assay^24^.

The pro-survival BCL-2-like proteins normally associate with the lipid bilayer of mitochondrial, endoplasmic reticulum (ER) and nuclear envelope membranes via their hydrophobic domains (Fig. 1a)^25^. Accordingly, confocal microscopy and cell fractioning have revealed that BCL-w associates with intracellular membranes^26^, and these interactions are strengthened under stress^27^. It has been demonstrated that in unstressed cells the C-terminal domain of BCL-w is folded back within the hydrophobic pocket, and remains only loosely attached to the mitochondrial membrane. When an apoptotic signal is received, C-terminal arm of BCL-w is released by a ligation of pro-apoptotic BH3-only protein, which consequently promotes a tight interaction between BCL-w and mitochondrion^28–30^. Notably, it has been demonstrated that the membrane-inserted pool of BCL-w interacts with BH3-only proteins, whereas BCL-w molecules loosely attached to the mitochondrial membrane are associated with MOMP-inducing proteins^28^. Deletion of C-terminal α-helix increased BCL-w binding affinity for BID-derived BH3 peptide, which indicates that this helix modulated interactions of BCL-w with pro-apoptotic partners by competing for peptide binding to the hydrophobic pocket^27^. More recent study that involved BCL-w in complex with designed ankyrin repeat proteins (DARPins) has revealed, however, greater structural similarity of BCL-w to ligand-free BCL-X_L_ than it was primarily thought^31^. In addition, the BCL-X_L_ C-terminus has also been shown to interact with a hydrophobic groove in the water-soluble form of the protein, however, the C-terminal tail in BCL-X_L_ did not trigger a conformational change and did not contribute to the formation of a tightly bound structure as observed in BCL-w^32^. It has been suggested that increased flexibility of the BCL-w groove area is not determined by the hinge regions, but by the weaker interactions between the α3-α4 and the α5-α6 helical hairpins of BCL-w^31^. Consequently, crucial interactions identified in the ligand-binding area of BCL-X_L_ are weakened or lost in BCL-w^31^, which is in line with previous observations showing weaker interactions between the BH1 domain of BCL-w and BID or BIM in comparison to BCL-X_L_/BID and BCL-X_L_/BIM complexes^33^. Identification of BCL-w homodimer has further envisaged a high conformational flexibility of BCL-w. The X-ray crystallography structure has revealed that helices α3 and α4 hinge away from the core of one molecule to cross into another BCL-w protomer. This conformation results in the dimerization-specific exposition of helices α5 and α6 while remaining BH3-binding pocket intact. BCL-w homodimer retains selectivity of binding to BH3-only proteins, but the affinity is lower than for monomeric BCL-w as exemplified for BAD binding with *K*_D_ = 150 nM and *K*_D_ = 14 nM, respectively^34^. Further research is necessary to delineate how the conformational flexibility of BCL-w is unique compared with other members of the BCL-2 family of proteins, and how it can be exploited in the development of the BCL-w-selective inhibitors.

## Regulation of BCL-w level

**Fig. 2:**
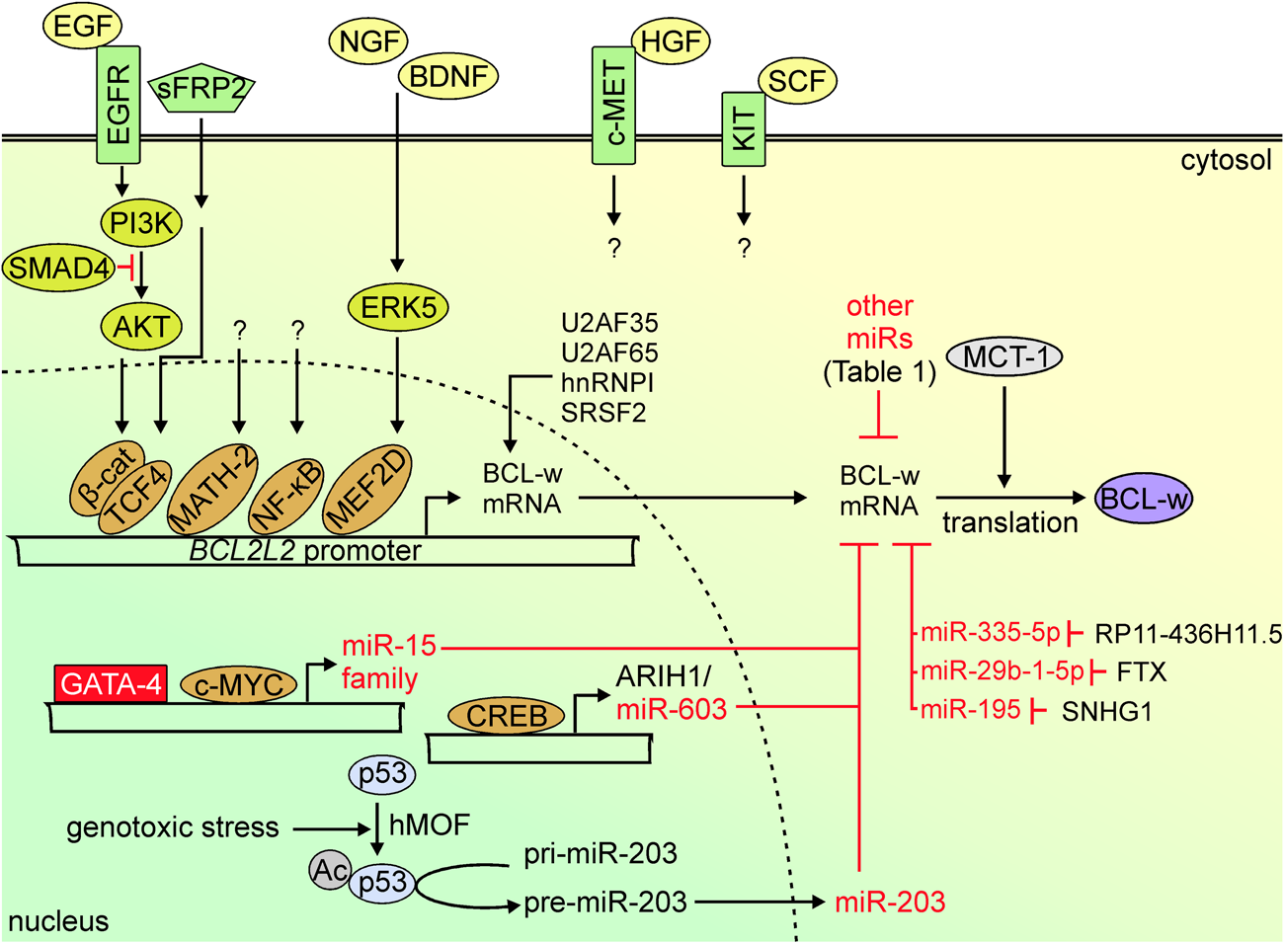
Signal transducers and effector transcriptional regulators involved in the control of *BCL2L2* expression. For HGF/c-MET and SCF/KIT signaling, no downstream elements specifically involved in the regulation of BCL-w level were demonstrated. In addition, a few transcription factors were shown to either activate (c-MYC, CREB, p53) or repress (GATA-4) expression of particular miRNAs (miRs) involved in the regulation of BCL-w mRNA level. Other miRs involved in the control of BCL-w level are extensively characterized in Table 1.

BCL-w protein, 193-amino acid residues in length (Fig. 1a) is encoded by *BCL2L2*, which is located on human chromosome 14 at band q11.2-q12^35^. *BCL2L2* consists of two coding exons in addition to two non-coding exons located at the 5′-end^36^. The *BCL2L2* promoter is highly conserved between human, mouse and rat, and the minimal promoter region lies within the non-coding exon 1a^37^. Analysis of the rat *Bcl2l2* promoter by using phylogenetic approach has revealed putative binding sites for several transcription factors including myocyte enhancer factor 2 (MEF2), erythroblastosis virus E26 oncogene homolog (ETS-1 and ETS-2), CCAAT/enhancer binding protein (C/EBP) and nuclear factor-kappa B (NF-κB)^37^. A number of signaling pathways and downstream transcription factors have been experimentally validated as the regulators of *BCL2L2* expression (Fig. 2), although the contribution of different transcriptional factors largely depends on the cellular and developmental context. *BCL2L2* was identified as a target of p65/NF-κB in chronic lymphocytic leukemia cells, which was confirmed in experiments involving BAY110782, an inhibitor of NF-κB^38^. In addition, p65/p52 NF-κB dimer was involved in upregulation of BCL-w in glial-cell-line-derived neurotrophic factor (GDNF)-treated dopaminergic neurons^39^. *BCL2L2* transcription was also positively regulated by the β-catenin/transcription factor 4 (TCF4) complex, and overexpression of either dominant-negative TCF4 (TCF4ΔN) or wild-type β-catenin resulted in decreased or increased activity of the *BCL2L2* promoter, respectively^36^. The role of secreted Frizzled-related protein 2 (sFRP2) in β-catenin-dependent expression of *BCL2L2* has also been reported^40^. Increased *BCL2L2* transcription was assessed after stimulation of distal axon with nerve growth factor (NGF) and brain-derived neurotrophic factor (BDNF), used either alone or in combination, and engaged ERK5-dependent phosphorylation of MEF2D transcription factor^41^. In addition, BCL-w was the only anti-apoptotic protein regulated by neuronal differentiation 6 factor (NeuroD6/MATH-2) under non-stress conditions, and NeuroD6/MATH-2 also assisted in a proper subcellular localization of BCL-w upon serum deprivation^37^. A positive correlation between expression of *BCL2L2* and *MET w*as demonstrated, and c-MET downregulation was followed by a decrease in mRNA level of BCL-w, but not other pro-survival members of the BCL-2 family^42^. Different reports have suggested a cell type-specific contribution of cAMP responsive element binding protein (CREB) to *BCL2L2* expression^43–45^. A temporal increase of CREB activity in adult visual neocortex was concomitant with an upregulation of anti-apoptotic molecules, including BCL-w^43^. In rat Sertoli cells, CREB was dispensable for 17-beta-estradiol-induced BCL-w expression^44^. In turn, CREB indirectly reduced the BCL-w level in colorectal cancer cells by binding to the promoter of the gene encoding ariadne RBR E3 ubiquitin protein ligase 1 (ARIH1), which contains *microRNA-603* (*miR-603)* within its exon^45^. A few other transcription factors can also indirectly control BCL-w level by affecting expression of miRs involved in BCL-w downregulation (Fig. 2). GATA-binding protein 4 (GATA-4) inhibited expression of miRs from the miR-15 family, including miR-15b, miR-16 and miR-195, and consequently promoted BCL-w-dependent survival of mesenchymal stem cells^46^. On the contrary, c-MYC upregulated the miR-15 family members responsible for the suppression of BCL-w expression, and this effect was independent of p53^47^. An indirect role of p53 in the control of BCL-w level was, however, demonstrated during genotoxic stress as a human ortholog of males absent on the first (hMOF)-mediated acetylation of Lys120 residue in p53 was essential for p53-dependent processing of miR-203, which downregulated BCL-w level^48^.

A number of other miRs were identified as negative regulators of BCL-w level by binding to the 3’-untranslated region (3’-UTR) of BCL-w transcript (Table 1). In addition, long non-coding RNA (lncRNA) RP11-436H11.5, which functions as a competitive endogenous RNA, was able to sponge miR-335-5p and in turn upregulate BCL-w level^49^. The sponging activity was also demonstrated for lncRNA FTX, which controlled miR-29b-1-5p-dependent BCL-w transcript level in mouse cardiomyocytes^50^, while a lncRNA small nucleolar RNA host gene 1 (SNHG1) sponged miR-195 in human cardiomyocytes^51^. Several splicing- and translation-regulating factors have been involved in the processing of BCL-w mRNA. Upstream sequence element (USE) in the 3’-UTR of BCL-w transcript contributed to 3’-end formation via interaction with splicing factors: U2 small nuclear RNA auxiliary factor 1 (U2AF35), U2 small nuclear RNA auxiliary factor 2 (U2AF65) and heterogeneous nuclear ribonucleoprotein I (hnRNPI)^52^. In addition, downregulation of serine and arginine-rich splicing factor 2 (SRSF2) was associated with decreased BCL-w transcript level^53^. It was also demonstrated that multiple copies in T-cell lymphoma-1 (MCT-1) protein interacted with the translation machinery to augment the translation of several mRNAs, including BCL-w transcript^54^.

**Table 1:** miRNAs (miRs) down-regulating BCL-w level. miRs and cell lines involved in experiments that directly evidenced the binding of particular miR to 3’-UTR of BCL-w transcript are included.

In vitro			
miR	Cell line	Cell type	Reference
let-7a-3p	U251 and A172	Glioblastoma multiforme	^107^
miR-15a	A549	Non-small cell lung cancer	^162^
	FaDu	Hypopharyngeal squamous cell carcinoma	^163^
miR-15b	SNU475	Hepatocellular carcinoma	^164^
miR-16	SCC-25	Oral squamous cell carcinoma	^165^
miR-29a	HEC-1B	Human endometrial carcinoma	^166^
miR-29b	HEK293	Human embryonic kidney epithelial cells	^76^
	U251 and U87MG	Glioblastoma multiforme	^141^
miR-29b-1-5p	-	Mouse cardiomyocytes	^50^
miR-29c	A549	Non-small cell lung cancer	^167^
miR-34a-5p	HEK293	Human embryonic kidney epithelial cells	^168^
miR-92a	H9c2	Rat cardiomyocytes	^169^
miR-93-5p	U251	Glioblastoma multiforme	^152^
	H460	Lung cancer	
miR-107	A549/Taxol	Paclitaxel-resistant non-small cell lung cancer	^110^
	HT-22	Immortalized hippocampal neuron cells	^170^
miR-122	Hep3B and HepG2	Hepatocellular carcinoma	^171^
	Mahlavu	Hepatocellular carcinoma (gefitinib-resistant)	^172^
	HeLa	Cervical cancer	^173^
	Huh7, Hep3B and HepG2	Hepatocellular carcinoma	
	H460	Lung cancer	
	HepG2	Hepatocellular carcinoma	^174^
	-	Pterygium epithelial cells	^88^
miR-125a-5p	PLC/PRF/5	Hepatocellular carcinoma	^175^
miR-125b	-	Rat hippocampal and cervical neurons	^176^
	SMMC7721	Hepatocellular carcinoma	^177^
miR-126-5p	SiHa and HeLa	Cervical cancer	^178^
miR-129-2-3p	MDA-MB-231	Breast cancer	^179^
miR-133b	T24	Bladder cancer	^115^
	U2OS and MG63	Osteosarcoma	^180^
	H2009	Non-small cell lung cancer	^181^
miR-146-5p	SKOV3	Ovarian cancer	^182^
miR-150	SKpac	Paclitaxel-resistant ovarian cancer	^183^
oxi-miR-184	H9c2	Rat cardiomyocytes	^184 ^^a^
miR-195	BEL7402	Hepatocellular carcinoma	^130^
	BEL7402/5-FU	Hepatocellular carcinoma (5-FU-resistant)	
	HT29 and LOVO	Colon cancer	^101^
	HCM	Human cardiomyocytes	^51^
miR-203	HEK293	Human embryonic kidney epithelial cells	^114^
	HCT116	Colon cancer	^48^
	T24 and 5637	Bladder cancer	^116^
	SGC-7901	Gastric cancer	^185^
miR-204	HTM1073 and HTM681	Human trabecular meshwork cells	^186^
miR-205	WPE1-NA22 and WPE1-NB26	Prostate cancer	^126^
	H460	Lung cancer	^103 ^^b^
	MDA-MB-231	Breast cancer	
miR-206	MCF-7 and TY7D	Breast cancer	^108 ^^b^
miR-214	HeLa	Cervical cancer	^120^
	HEK293	Human embryonic kidney epithelial cells	^187^^ c^
miR-336	SKOV3 and ES2	Ovarian cancer	^140^
	SGC-7901	Gastric cancer	^143^
	A549 and H1299	Non-small cell lung cancer	^109^
	786-O and CaKi-1	Clear cell renal carcinoma	^119^
	A2780	Ovarian cancer	^127 ^^b^
	A2780/DDP	Ovarian cancer (cisplatin-resistant)	
	A498 and 786-O	Renal cell carcinoma	^49^^ b^
miR-340-5p	U251 and U87	Glioblastoma multiforme	^188^
miR-378	SKM-1	Acute myeloid leukemia	^189^
miR-422a	U2OS and MG63	Osteosarcoma	^123^
mmu-miR-466h	CHO	Mammalian Chinese hamster ovary	^190^
miR-497	N2A	Mouse neuroblastoma	^74^
	MCF-7	Breast cancer	^191^
miR-509-3p	HEK293T	Human embryonic kidney epithelial cells	^192^
miR-630	JHU-029	Head and neck squamous cell carcinoma	^133^
	SW480 and HT29	Colorectal cancer	^45^

**In vivo**			
**miR**	**Cell type**	**In vivo** **experimental model**	**Reference**
miR-15a	Neuronal cells	miR-15a/16-1^null^ mice	^75^
miR-16	Oral squamous cell carcinoma	BALB/c nude mice	^166^
miR-92a	Rat cardiomyocytes	Sprague–Dawley rats	^169^
miR-107	Paclitaxel-resistant non-small cell lung cancer	Nude mice	^110^
miR-122	Gefitinib-resistant hepatocellular carcinoma	BALB/c nude mice	^174^
miR-205	Lung cancer and breast cancer	BALB/c nude mice	^103^^ b^
miR-336	Gastric cancer	BALB/c nude mice	^143^
	Cisplatin-resistant ovarian cancer	BALB/c nude mice	^127 ^^b^
	Renal cell carcinoma	Nude mice	^49^
miR-378	Acute myeloid leukemia	NOD/SCID mice	^189^
miR-497	Neuronal cells	C57/B6 mice	^74^

^a^ROS-mediated oxidative modification of miR-184 (oxi-miR-184) is indispensable for recognition of BCL-w mRNA.

^b^5p form of miR was used.

^c^3p form of miR was used.

## Role of BCL-w in normal cells and non-cancer diseases

BCL-w has been already detected in a number of solid tissues, including testes, colon, and brain, as well as in cells of myeloid and lymphoid origin^26,35^. Mice that lacked BCL-w were viable, exerted normal appearance and most of their tissues exhibited typical histology. However, the males were infertile in contrast to female mice that could efficiently reproduce. It was observed that the seminiferous tubules of BCL-w-deficient male mice contained apoptotic cells, and the numbers of both Sertoli cells and germ cells were reduced^55,56^. Further studies confirmed the essential contribution of BCL-w to spermatogenesis^26^, and demonstrated that BCL-w was largely expressed in Sertoli cells^57,58^, Leydig cells, spermatogonia, and spermatocytes^57^. Elevated levels of BAX/BCL-w and BAK/BCL-w complexes were found in most of these types of cells^57^ suggesting a functional significance of BCL-w in their survival. Accordingly, BCL-w promoted survival of mouse post-mitotic Sertoli cells by suppressing BAX-dependent apoptotic activity^59^. It was demonstrated that BCL-w-dependent survival of germ cells was regulated by stem cell factor (SCF), which simultaneously downregulated expression of pro-apoptotic members of the BCL-2 family, including BAX^60^, while decreased BCL-w protein levels were assessed in testes from cigarette smoke-exposed rats^61^. Interestingly, BCL-w overexpression impaired spermatogenesis as it prevented from entering cell cycle^62^. Testes of transgenic mice that overexpressed BCL-w exhibited degeneration of spermatocytes, vacuolization of Sertoli cells and reduced number of spermatogonia^62^. This indicates that temporal and spatial expression of *BCL2L2* can be essential for normal development and function of testes.

BCL-w has also been shown to contribute to survival of epithelial cells in the gut^26^. BCL-w protected small intestine- and midcolon-derived epithelial cells from apoptosis induced either by 5-fluorourcil (5-FU) or gamma-irradiation, although spontaneous cell death was not substantial upon loss of BCL-w in these cells^63^. In addition, BCL-w promoted enterocyte survival after massive small bowel resection, and the role of epidermal growth factor (EGF) was implicated in this process^64^. The activation of epidermal growth factor receptor (EGFR) decreased BAX/BCL-w ratio, which shifted the balance to cell survival^64–66^. Accordingly, poor survival and impaired adaptation after the resection of small bowel were observed in either BCL-w^null^ or EGFR-deficient mice^66^ suggesting that manipulation of EGF-EGFR-BCL-w pathway might be therapeutically relevant in patients after massive resection of small intestine.

A stage-dependent increase in BCL-w transcript level has been reported during the development of rat brain^67^. The high levels of BCL-w were assessed in several regions of the mature brain, including cerebellum, hippocampus, and sensory neurons, whereas BCL-X_L_ was abundantly expressed during early stages of development^67,68^. At the molecular level, both serine and glycine could selectively upregulate *BCL2L2* expression in neuronal cells, while retaining BCL-X_L_ level unaltered^69^. BCL-w also controlled the mitochondria morphogenesis and dendrite development in Purkinje cells, and was involved in synapse formation in mouse cerebellum. In this context, BCL-w did not determine number of cells in the brain, but promoted mitochondrial fission in Purkinje dendrites, which was also shown in vivo as BCL-w^null^ mice displayed a marked increase in mitochondrial length^70^. The role of BCL-w has also been demonstrated in several disorders of neuron functions and neurodegenerative diseases. Increased expression of BCL-w was found in ischemic brain suggesting a neuroprotectant role of this protein^71,72^. Accordingly, study on the rat model revealed that overexpression of BCL-w significantly improved neurological functions after focal cerebral ischemia in up to 40% animals^73^. In this respect, also indirect manipulation of BCL-w level could attenuate ischemic damage of the brain as exemplified by inhibition of miR-497 (Table 1), which was involved in downregulation of BCL-w and neuronal death after ischemia^74^. In addition, upregulation of BCL-w as a result of intravenous delivery of miR-15a/16-1 antagomir or *miR-15a/16-1* knockout, reduced size of cerebral infarct and improved sensorimotor deficits in a middle cerebral artery occlusion (MCAO) mice^75^. Using a rat model of transient MCAO and oxygen-glucose deprivation in neurons, it was demonstrated that miR-29b contributed to cell death following ischemic injury as it inhibited BCL-w^76^. Protein level of BCL-w was also affected in the hippocampus after seizures^77^. BCL-w was upregulated following brief electroshock seizures, whilst it was bound to BIM and integrated in the mitochondrial membrane in damaged subfields after *status epilepticus*^77^. Moreover, epileptic seizures induced more significant nuclear fragmentation and hippocampal damage in BCL-w-deficient mice compared with wild-type controls^77^. In addition, an increased BCL-w protein level associated with punctate intracytoplasmic structures was found in a model of Alzheimer’s disease, in contrast to low level and diffuse distribution of BCL-w in control cases^78^. Mechanistically, it was shown that overexpression of BCL-w protected neurons from β-amyloid-induced cell death by blocking mitochondrial release of Smac, as accumulation of β-amyloid has been proposed as a key factor of neuron loss in Alzheimer’s disease^78,79^. In turn, β-amyloid reduced BCL-w protein level via c-JUN N-terminal kinase (JNK)-dependent mechanism^79^, whilst hyperactivation of AKT could counteract β-amyloid-mediated downregulation of BCL-w and cytotoxicity^80^. Neurotoxicity of β-amyloid was substantially attenuated through manipulation of the BCL-w level by β-asarone, a natural compound isolated from *Acorus tatarinowii Schott* (Table 2)^81,82^. *BCL2L2* expression was also significantly higher in Parkinson’s disease patient-derived dopaminergic neurons harboring mutant *PARK2*^83^. In turn, *BCL2L2* was hypermethylated and expressed at lower levels in multiple sclerosis-affected brain samples than in controls^84^. The role of BCL-w was also implicated in the viability of nociceptors as BCL-w knockout mice developed the symptoms of small fiber sensory neuropathy, including a decline in sensitivity to thermal stimuli and reduced innervation within the epidermis^85^. BCL-w level was increased in axons of sensory neurons, and cells deprived of BCL-w exerted mitochondrial dysfunctions such as abnormal size and membrane potential, and low level of intracellular ATP^85^. A forkhead box O3 (FOXO3a)/c-JUN-dependent upregulation of PUMA followed by inhibition of BCL-w was necessary to initiate axon degeneration^86^. More recently, it was shown that BH4 domain of BCL-w interacted with inositol 1,4,5-trisphosphate receptor 1 (IP_3_R1) and protected axons from degeneration^87^. This cytoprotective mechanism could be impaired by chemotherapeutics used in the treatment of cancer patients as shown for paclitaxel. Paclitaxel diminished the level of RNA-binding protein splicing factor proline and glutamine rich (SFPQ) and reduced translation of BCL-w transcript. As a consequence, deregulation of IP_3_R1 triggered neuronal degeneration associated with mitochondrial dysfunction and calpain-dependent proteolysis, which largely contributed to the chemotherapy-induced peripheral neuropathy^87^.

**Table 2:** Drugs and natural compounds (^N^) that affect BCL-w level.

Drug or compound	Disease model (cell line)	Effective concentration	Effect on BCL-w level	Level of regulation	Reference
β-asarone^N^	β-amyloid-induced rat model of Alzheimer’s disease	12.5 mg/kg	Up	mRNA protein	^81^
		25 mg/kg			
		50 mg/kg			
	β-amyloid-treated rat pheochromocytoma (PC12)	15 µg/ml	Up	mRNA protein	^82^
Curcumin^N^	Breast cancer (MCF-7)	25 µg/ml	Up	mRNA	^193^
		50 µg/ml			
	Breast cancer (MCF-7 and MDA-MB-231)	5 µM	No effect	protein	^194^
Genistein^N^	β-amyloid-treated rat pheochromocytoma (PC12)	25 µM	Up	mRNA	^195^
Phenethyl isothiocyanate (PEITC)^N^	Sprague–Dawley rats (hepatic cells)	150 µmol/kg	Up	mRNA	^196^
Cisplatin	HNSCC (JHU-029)	10 µg/ml	Down	protein	^133^
*Coptidis Rhizoma* extract^N^	Melanoma (A2058, UACC257 and UACC62)	100 µg/ml	Down	mRNA protein	^197^
Cyramza (ramucirumab)	NSCLC (HCC4006)	nd.	Down	protein	^111^
Dihydromyricetin ^N^	NSCLC (A549 and H1975)	75 µM	Down	protein	^198^
Fisetin^N^	HCC (Huh-7)	60 µM	Down	protein	^199^
Isoledene^N^	Colorectal cancer (HCT-116)	8–28 µg/ml	Down	mRNA	^200^
Phenazine-1-carboxamide (PCN)^N^	Breast cancer (MDA-MB-231) NSCLC (A549)	nd.	Down	mRNA protein	^201^
Sanguinarine^N^	N-Myc-negative neuroblastoma (SH-SY5Y)	5 µM	Down	mRNA	^202^
Tanshinone IIA^N^	Ovarian cancer (A2780)	150 µM	Down	protein	^203^
Quercetin^N^	Mouse neuroblastoma (N2a)	20 µM	Down	mRNA	^204^
		40 µM			

*HCC* hepatocellular carcinoma, *HNSCC* head and neck squamous cell carcinoma, *NSCLC* non-small cell lung cancer, *nd.* not determined.

Several reports have revealed the putative contribution of BCL-w in other types of cells. Abundant expression of *BCL2L2* was found within the whole epithelium and blood vessels of pterygium, in contrast to the presence of BCL-w protein predominantly in the basal layer of epithelium in normal conjunctiva^88^. Recently, it was also shown that *BCL2L2* overexpression contributed to the survival of megakaryocytes and increased formation of platelets^87^. A positive correlation between platelet numbers and BCL-w transcript levels in platelets was assessed in 154 healthy donors^89^. A fundamental role of BCL-w was also reported in the survival of B lymphocytes, as a loss of BCL-w substantially accelerated cell death upon deprivation of growth factors^47^. It was also demonstrated that BCL-w prevented from osteogenic differentiation of human mesenchymal stem cells^90^.

## Contribution of BCL-w to survival of cancer cells and their response to anti-cancer drugs

Elevated level of BCL-w has been assessed in various types of cancers^91^, but survival of different types of cancer cells does not predominantly rely on BCL-w as exemplified for acute myeloid leukemia^92^ and melanoma^93^. Only few genetic alterations of *BCL2L2* have been detected in cancers, including copy-number variations in small^94^ and non-small^95^ cell lung cancer, and a 3’-UTR variant (rs1950252) that was significantly associated with the risk of oral cancer^96^. In a large-scale analysis of somatic copy-number alterations, *BCL2L2* has been, however, classified as neither deleted nor amplified across different types of human cancers^97^. This suggests that increased level of BCL-w is rather a consequence of abnormal activation of cancer-related signaling pathways, and BCL-w cooperates with oncogene activation in development and progression of cancer.

A significantly higher level of BCL-w was assessed in gastric adenocarcinomas compared with normal neighboring mucosa, and BCL-w was associated with infiltrative morphotype of the tumor^98^. BCL-w level was also associated with poor survival of patients with colorectal cancer^99^. BCL-w was expressed at low levels in colorectal adenomas, while the majority (92%) of adenocarcinomas showed positive staining for BCL-w^100^ suggesting the contribution of BCL-w to cancer progression. This was also supported by higher BCL-w level in samples with node involvement, and in TNM stage III tumors compared with TNM stage II specimens^100^. BCL-w inhibited cell apoptosis by precluding activation of stress-activated protein kinase (SAPK)/JNK in gastric cancer cells^98^. A high level of BCL-w in colorectal cancer cells was related to a loss of SMAD family member 4 (SMAD4)^99^. Downregulation of BCL-w increased ionizing radiation (IR)-induced cytotoxicity in human colorectal cancer cell lines^45^. BCL-w conferred resistance to 5-FU^99^. BCL-w protein level was also increased in doxorubicin-resistant colon cancer cells, while the BCL-w inhibition partly reversed resistant phenotype^101^.

Hypomethylation status of *BCL2L2* was frequently observed in patients with glioblastoma multiforme (GBM), which exerted a high proliferation index and low sensitivity to apoptosis^102^. Consequently, expression of *BCL2L2* was significantly higher in GBM than in low-grade gliomas^103,104^, and BCL-w was involved in an aggressive phenotype of glioblastoma cells associated with specificity protein 1 (Sp1)-dependent expression of stem cell-related markers^104^. In addition, conditioned medium from the culture of BCL-w-overexpressing cells promoted tumorigenicity of GBM, which was associated with elevated levels of SRY-box 2 (SOX-2), NANOG, octamer-binding transcription factor 4 (OCT4), Nestin, NOTCH2, Musashi and CD133^103^. Increased BCL-w level was accompanied with upregulation of platelet-derived growth factor alpha (PDGFα)^103^. BCL-w overexpression also promoted formation of neurospheres^105^. BCL-w was required for tumor necrosis factor-like weak inducer of apoptosis (TWEAK)-dependent protection of glioblastoma cells against TRAIL and camptothecin^106^. Downregulation of BCL-w accompanied neurotensin receptor-1 (NTSR1) inhibition-induced mitochondrial apoptosis in glioblastoma cells, while restoration of BCL-w expression rescued these cells to certain extent^107^.

BCL-w mRNA level was also significantly higher in breast cancer specimens than in adjacent normal cells^103,108^. In addition, the level of BCL-w transcript was higher in plasma of patients with metastatic disease compared to that of patients with primary tumors^103^. BCL-w facilitated proliferation of breast cancer cells through a mechanism involving lncRNA HOX transcript antisense RNA (HOTAR)-dependent sequestration of miR-206, which downregulated BCL-w^108^. Moreover, BCL-w was implicated in resistance of breast cancer cells to radiotherapy. BCL-w was induced in response to IR via a mechanism involving hypermethylation of CpG islands within *miR-205-5p* promoter, which resulted in the upregulation of *BCL2L2* in both in vitro and in vivo models^103^. IR-induced BCL-w contributed to mesenchymal traits of cancer cells, and supported different phenotypes, including angiogenic, migratory, and stem cell-like phenotype^103^.

BCL-w promoted survival of non-small cell lung cancer cells^109^, and its overexpression was significantly associated with advanced tumor stage^95^. BCL-w level was higher in paclitaxel-resistant than in paclitaxel-sensitive non-small cell lung cancer cells, and miR-107-dependent downregulation of BCL-w sensitized resistant cells to the drug^110^. BCL-w level also determined the extent of lung cancer cell response to cyramza, a drug used for inhibition of vessel formation^111^. This might be related to the role of BCL-w in tumor angiogenesis. It was demonstrated in a mouse model of melanoma that blood vessel formation was enhanced upon interactions between endothelial cells (ECs) and pericytes as pericytes promoted EC survival via paracrine integrin α_V_- and NF-κB-dependent regulation of gene expression in endothelial cells, including BCL-w^112^. In addition, knockdown of BCL-w increased sensitivity of melanoma cells to tetrathiomolybdate (TTM), which is a copper chelator^113^.

BCL-w has been associated with malignancy of urinary system. BCL-w protein level was substantially higher in bladder tumor cells than in adjacent normal cells^114^, which was also confirmed in a cohort of 41 bladder cancer samples^115^. High level of BCL-w accompanied bladder cancer progression, and downregulation of BCL-w sensitized cells to cisplatin^116^. Notably, BCL2L2-PABPN1 chimeric RNA, which was generated by cis-splicing of adjacent genes, was detected at significantly higher level in bladder cancer specimens than in normal cells. Additionally, BCL2L2-PABPN1 RNA was preferentially detected in the nuclear fraction suggesting the role as a lncRNA^117^. BCL-w also showed significantly higher expression in metastatic clear cell renal cell carcinoma than in primary tumor cells^118^, which is consistent with the study demonstrating that overexpression of BCL-w increased the proliferation rate and invasion of these cancer cells^119^.

The role of BCL-w has been also implicated in survival of other types of cancers and their response to drugs. Expression of *BCL2L2* was significantly higher in cervical tumor samples compared with normal cervix tissue^120^. Downregulation of BCL-w reduced cell survival and attenuated resistance of cervical cancer cells to cisplatin^120^, and accelerated paclitaxel-induced mitotic cell death in vitro^121^. *BCL2L2* was selectively upregulated in samples of endometrial cancer representing G2 histological stage^122^. Downregulation of BCL-w enhanced serum deprivation-induced apoptosis in osteosarcoma cells^123^, while increased level of BCL-w protein accompanying overexpression of miR-196a promoted survival of osteosarcoma cells in vitro^124^. A significantly higher BCL-w protein level was also assessed in leiomyosarcomas in comparison to benign uterine smooth muscle tumors, and BCL-w expression reversely correlated with overall patient survival^125^. BCL-w protein level was also increased in advanced prostate cancer cell lines, which might result from epigenetic silencing of *miR-205* expression, and conferred resistance to cisplatin and docetaxel^126^. In addition, a high expression of BCL-w rendered resistance of ovarian cancer cells to cisplatin, and BCL-w knockdown significantly reduced size of tumor derived from cisplatin-resistant cells^127^. Downregulation of *BCL2L2* re-sensitized ovarian cancer cells resistant to etoposide (VP-16)^128^. Recently, *BCL2L2* has been correlated with drug resistance of high-grade serous ovarian cancer (HGSOC) cells^129^. BCL-w protein level was also markedly higher in hepatocellular carcinoma (HCC) cells resistant to 5-FU compared with matched drug-sensitive cells^130^. miR-122-dependent downregulation of BCL-w rendered HCC cells sensitive to adriamycin and vincristine^131^, while inhibition of BCL-w and BCL-2 as a result of cyclooxygenase-2 (COX-2) silencing potentiated TRAIL-mediated apoptosis in HCC cells^132^. In head and neck squamous cell carcinoma cells, cisplatin-induced miR-630-dependent downregulation of BCL-w was reported^133^. A high expression of *BCL2L2* was assessed in diffuse large B-cell lymphoma (DLBCL) and in almost 90% of patients with Burkitt lymphoma (BL). BCL-w knockdown induced apoptosis in Burkitt lymphoma cells whilst BCL-w overexpression conferred resistance to ABT-737 and ABT-263, BH3 mimetics targeting BCL-2-like proteins^47^. Downregulation of BCL-w markedly delayed MYC-mediated development of B-cell lymphoma^47^. In another report, however, BCL-w was expressed at high level only in a subset of BL and DLBCL cell lines. Moreover, CRISPR/CAS9 gene editing or RNA interference leading to downregulation of *BCL2L2* expression did not sensitize lymphoma cells to apoptosis, even when these cells were exposed to BH3 mimetics^134^. It has been also demonstrated that BCL-w, in addition to BCL-2 and BCL-X_L_, played a minor role in the development of sarcoma and thymic lymphoma in *p53*-deficient mice^135^. BCL-w was highly expressed in patient-derived B-cell chronic lymphocytic leukemia (B-CLL) cells in comparison to normal peripheral blood lymphocytes^136^. BCL-w was also involved in autocrine exosome-mediated regulation of chronic myeloid leukemia cell survival^137^.

## Role of BCL-w in migratory and invasive potentials of cancer cells

**Fig. 3:**
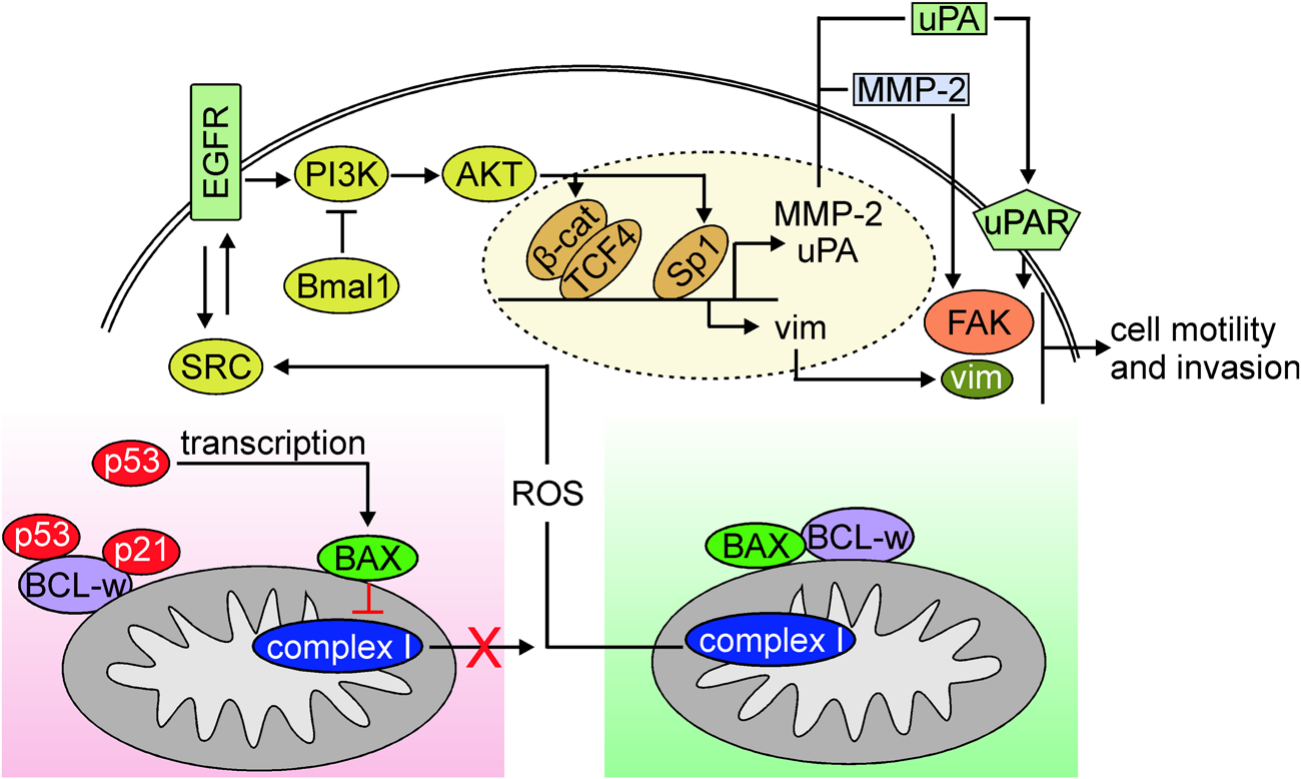
BCL-w controls cell motility. Cell motility and invasion are promoted by BCL-w-mediated inhibition of BAX that enables the mitochondrial complex I to produce ROS (green background). ROS activate signaling cascade involving SRC-EGFR-PI3K/AKT, which engages transcriptional regulators, β-catenin/TCF4 and Sp1, to upregulate expression of genes encoding pro-invasive and pro-migratory molecules, including MMP-2, uPAR, and vimentin (vim). On the contrary, cell motility and invasion are attenuated upon substitution of BAX for cytosolic p53 and p21 in the BAX/BCL-w complex (red background). BAX resides in the outer mitochondrial membrane, and the topology allows C-terminal four residues of BAX (KKMG) to interact with a subunit of complex I in the inner membrane of mitochondria, which is followed by inhibition of ROS production. BAX-dependent attenuation of ROS synthesis is potentiated by nuclear p53-driven transcription of *BAX* that contributes to increased BAX protein abundance.

Pro-survival proteins from the BCL-2 family have been shown to contribute to migratory and invasive capabilities of normal and cancer cells^138,139^, and the role of BCL-w to this process has been delineated. It was reported that ectopic *BCL2L2* expression almost fully nullified the inhibitory effect of miR-335 on migration and invasion of ovarian cancer cells^140^. BCL-w potentiated mesenchymal phenotype of GBM cells^141,142^, and regulated the invasion capability of human gastric cancer cells^143^. BCL-w enhances the migratory and invasive potentials of gastric cancer cells by facilitating the production of several types of extracellular matrix (ECM)-degrading proteinases^126^. Secreted matrix metallopeptidase-2 (MMP-2) and urokinase plasminogen activator surface receptor (uPAR) have been demonstrated to activate focal adhesion kinase (FAK), which acts as an executioner of BCL-w-dependent invasive phenotype of gastric cancer cells^144^. Mechanistically, BCL-w increases the level of mitochondria-derived reactive oxygen species (ROS), which is followed by SRC-mediated phosphorylation of EGFR^145^, and the activation of PI3K/AKT/Sp1 signaling pathway to increase *MMP2* expression in GBM and gastric cancer cells^18,104,146^. BCL-w promotes activation of MMP-2 and FAK via PI3K/AKT/β-catenin signaling pathway in GBM cells^105,142^, while BCL-w-induced nuclear accumulation of β-catenin contributes to the upregulation of vimentin (Fig. 3)^141,142^. Notably, BCL-w-mediated BAX inhibition is essential for cell invasion as a variant of BCL-w (BCL-w^G94A^) that does not bind to BAX failed to stimulate ROS production and cell invasion^18^ as well as cancer cell intravasation in an in vivo model of lung cancer^147^.

On the contrary, several mechanisms to counteract BCL-w-dependent cell invasion and motility have been evidenced. The PI3K/AKT/MMP-2 signaling pathway involved in cell invasion-promoting activity of BCL-w is inhibited by brain and muscle aryl hydrocarbon receptor nuclear translocator (ARNT)-like (Bmal1) in GBM and lung cancer cells^148^. In addition, cytosolic p53 liberates BAX from BCL-w and suppresses non-small cell lung cancer cell invasion by attenuation of ROS production^147^. This is driven by BAX-dependent inhibition of NADH:ubiquinone oxidoreductase core subunit 5 (ND5), a subunit of respiratory complex I^147^. Simultaneously, nuclear p53 augments the pool of BAX molecules via executing transcription of *BAX*^147^. The inhibitory role of p21 in the regulation of BCL-w-dependent lung cancer, colon cancer, and neuroblastoma cell invasion has been demonstrated in addition to p53^149^. Although p53 and p21 can bind to BCL-w independently, the triple p53/p21/BCL-w complex is required for BAX release from BCL-w and suppression of cell invasion (Fig. 3)^149^.

## Role of BCL-w in cellular senescence

Cellular senescence is a form of cell cycle arrest that can develop in response to DNA damage, nutrient deficiency, telomere shortening, oxidative stress, and oncogene activation. Senescence induction is often executed as a barrier against tumorigenesis, but senescent cells can produce growth factors and cytokines, collectively named as the senescent-associated secretory phenotype (SASP), which can promote tumor development^150^. It was demonstrated that co-inhibition of BCL-w and BCL-X_L_ by specific siRNAs or by a BH3 mimetic (ABT-737) induced apoptosis in senescent human fibroblasts in vitro^151^. This observation was further validated in an in vivo model, and ABT-737 efficiently eliminated epidermal cells exhibiting senescent features triggered by DNA damage or p14^ARF^-p53 activation^151^. More recently, BCL-w contribution to senescent phenotype has also been evidenced in GBM and lung cancer cells^152^. BCL-w promotes senescence-associated β-galactosidase (SA-β-gal) activity and trimethylation of histone H3, as well as expression of genes encoding senescence-related proteins including p53, p21, and p16^152^. It has also been shown that overexpression of miR-93-5p in GBM and lung cancer cells is sufficient to prevent from premature senescence through downregulation of BCL-w and p21^152^.

## Concluding remarks and future perspectives

**Fig. 4:**
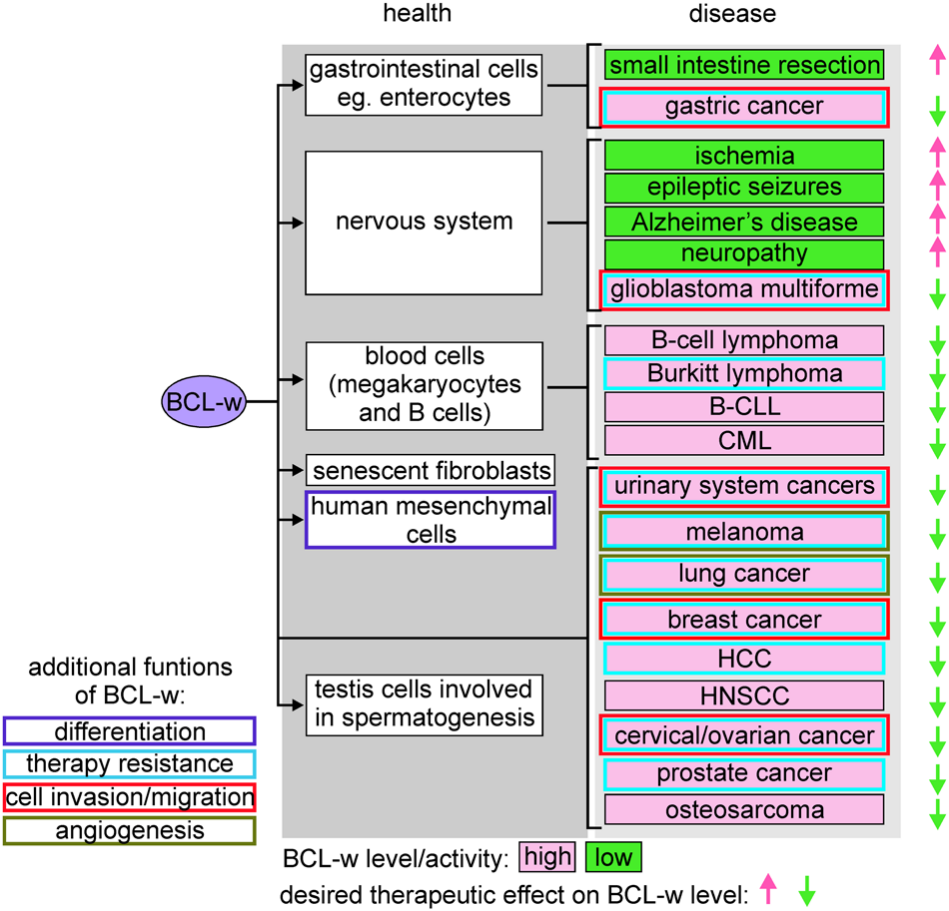
An overview of cell type-specific roles of BCL-w. BCL-w is broadly expressed in many types of normal cells as well as diseased cells, in which either increased (red background) or decreased (green background) BCL-w levels are assessed. In addition to the pro-survival role exerted in health and disease, BCL-w regulates additional cell programs and functions (color frames). Consequently, drugs that either decrease or increase BCL-w level and activity can exhibit therapeutic relevance against different disorders.

BCL-w with diverse functions in development, health, and disease, can play both positive and negative roles in the particular process or cellular context. BCL-w is an attractive therapeutic target as its inhibition might be relatively well-tolerated in patients. This is supported by studies showing that loss of BCL-w was associated with defects in spermatogenesis and small intestine cells in mice but had no deleterious effects in the majority of other tissues^56,58,63^. The contribution of BCL-w to differentiation of lymphocytes has appeared questionable as *BCL2L2*-knockout mice exhibited unaffected lymphoid development^55^, probably as a result of low level of BCL-w in normal and malignant lymphoid cells^26^. Further research is necessary to determine an unequivocal role of BCL-w in these cells in the light of conflicting results of more recent reports^47,134^. Notably, the redundant role of BCL-w is in sharp contrast to other pro-survival members of the BCL-2 family that have been shown essential during embryogenesis, development of nervous system and hematopoiesis as exemplified especially by BCL-2, MCL-1 and BCL-X_L_^153–155^. Thus, observations from experiments using knockout mice have provided an overview of the loss-of-function phenotypes that may have an impact on prediction of clinical applications of the drugs that inhibit activity of specific pro-survival proteins. Consequently, while tissue-specific BCL-w inhibition can be beneficial to overcome therapy resistance of cancer patients, increasing BCL-w level might be therapeutically relevant in a number of neurological disorders and after small intestinal resection (Fig. 4). In addition, the role of BCL-w in sustaining the survival of senescent cells suggests that manipulating BCL-w can be an useful approach in age-related disorders. To not disturb overall organismal homeostasis and limit unwanted drug cytotoxicity, it is essential to define actual cell dependence on specific anti-apoptotic protein eg., BCL-w. In this respect, BH3 profiling can be used to identify protein(s) that must be inhibited to efficiently execute MOMP^156^ while Dynamic BH3 profiling, which has been established more recently as an alternative functional approach, allows to measure cell dependence that can be altered in response to drugs^157^. For the time being, there are no drugs that selectively affect BCL-w level, which might be associated with a high conformational flexibility of this protein. Several drugs and natural compounds have been shown to affect BCL-w level in in vitro and in vivo models of different diseases (Table 2), however, BCL-w is not their exclusive target. Two BH3 mimetics, ABT-737 and its orally bioavailable derivative ABT-263, represent agents that inhibit BCL-w activity^5,158^. As both compounds mimic BAD, they neutralize BCL-2 and BCL-X_L_ in addition to BCL-w^5,159^. Moreover, it has been demonstrated that ABT-737 displaces BIM from BCL-w with much lower efficiency than from other pro-survival proteins^160,161^ suggesting that cellular effects induced by ABT-737/ABT-263 could predominantly result from BCL-2 and BCL-X_L_ inhibition. For that reason, further research directed to the development of selective drugs either upregulating or inhibiting BCL-w is still needed.
